# Supplementary material for: Seasonal Effect of Grass Nutritional Value on Enteric Methane Emission in Islands Pasture Systems
Source: Animals (Basel). 2023 Aug 30;13(17):2766. doi: 10.3390/ani13172766 (PMC10486569; doi:10.3390/ani13172766)
Supplement: Supplementary file 1 [file animals-13-02766-s001.zip › animals-2554801-supplementary.pdf]

**Table S1** - Productive characteristics of the animals under study

| Category | | BW (Kg) | Milk Production (Kg/day) | Anual Milk Production (Kg/head) | % Milk Fat | Average Daily Gain (Kg/day) |
|---|---|---|---|---|---|---|
| Calves | Beef Calves | 200 | n.a | n.a | n.a | 0,8 |
| | Dairy Calves Male | 175 | n.a | n.a | n.a | 0,6 |
| | Dairy Calves Female | 175 | n.a | n.a | n.a | 0,6 |
| Dairy cattle | Pregnant | 550 | 24 | 7320 | 3,6 | n.a |
| | Non-pregnant | 450 | 18 | 5490 | 3,6 | n.a |
| Beef cattle | Pregnant | 580 | 5,8 | 1769 | 3,8 | n.a |
| | Non-pregnant | 540 | 4,5 | 1372,5 | 3,8 | n.a |
| Replacement heifers | | 400 | n.a | n.a | n.a | 0,6 |
| Other bovines | | 550 | n.a | n.a | n.a | 1,2 |
| Breeding bulls | | 700 | n.a | n.a | n.a | 0 |

n.a – not applicable.
